# Cochlear synaptopathy and hidden hearing loss: a scoping review

**DOI:** 10.1590/2317-1782/20232023032en

**Published:** 2023-11-20

**Authors:** Marina de Figueiredo Colla, Pamela Papile Lunardelo, Fernanda Abalen Martins Dias

**Affiliations:** 1 Departamento de Fonoaudiologia, Pontifícia Universidade Católica de Minas Gerais – PUC MG - Belo Horizonte (MG), Brasil.; 2 Programa de Pós-graduação em Psicobiologia, Universidade de São Paulo de Ribeirão Preto – USP RP - Ribeirão Preto (SP), Brasil.

**Keywords:** Hearing, Hidden Hearing Loss, Cochlear Synaptopathy, Terminology, Review

## Abstract

**Purpose:**

To identify the pathophysiological definitions adopted by studies investigating “cochlear synaptopathy” (CS) and “hidden hearing loss” (HHL).

**Research strategies:**

The combination of keywords “Auditory Synaptopathy” or “Neuronal Synaptopathy” or “Hidden Hearing Loss” with “etiology” or “causality” or “diagnosis” was used in the databases EMBASE, Pubmed (MEDLINE), CINAHL (EBSCO), and Web of Science.

**Selection criteria:**

Studies that investigated CS or HHL in humans using behavioral and/or electrophysiological procedures were included.

**Data analysis:**

Data analysis and extraction were performed with regard to terminology, definitions, and population.

**Results:**

49 articles were included. Of these, 61.2% used the CS terminology, 34.7% used both terms, and 4.1% used HHL. The most-studied conditions were exposure to noise and tinnitus.

**Conclusion:**

CS terminology was used in most studies, referring to the pathophysiological process of deafferentiation between the cochlear nerve fibers and inner hair cells.

## INTRODUCTION

Cochlear synaptopathy (CS) is characterized by deafferentiation between cochlear nerve fibers and inner hair cells (IHC) in the spiral ganglion (SG). Cochlear neurons and their vulnerable synaptic connections are the main targets of some pathological agents, with predominant involvement of low spontaneous rates and high-threshold fibers^(1)^. This pathological process is extra-axial and precedes permanent changes in the auditory threshold. Thus, synaptic deterioration occurs even when the IHC remains intact^(2,3)^.

Over the years, different terminologies have been assigned to the auditory profile characterized by the presence of normal hearing and suprathreshold deficits^(4)^ because this manifestation may be associated with different diseases that affect the auditory system. Currently, the most common designations for deafferentiation are the CS and Hidden Hearing Loss (HHL).

The main clinical manifestations of CS are difficulty in understanding speech in noisy environments, tinnitus, and hyperacusis in the presence of normal hearing^(3,5-11)^. However, it should be emphasized that such manifestations are common to several auditory and/or otological pathological processes in addition to CS.

Different factors have been identified as the cause of CS, with exposure to noise and aging being the main factors^(1,3,4,8,12,13)^. Exposure to high sound pressure levels can cause damage between the synapses of the IHC and the nerve endings of the auditory nerve^(1,14)^, as it causes excessive release of glutamate in the postsynaptic receptor of the cochlear nerve, promoting excitotoxicity and swelling in the fiber terminals of the SG^(1,15)^ and initiating a degenerative cascade marked by a temporary increase in the auditory threshold, which is considered transitory^(1)^.

Aging is another possible causal factor. Based on the analysis of the temporal bone in postmortem studies^(16)^, loss of auditory nerve fibers is identified even in the absence of IHC death, or even more pronounced loss of these fibers when there is already cell death. Changes in the auditory threshold only occur when neuronal loss exceeds 80–90%^(2,17)^. Although this is the “gold standard” procedure to indicate CS, the quantification of cochlear synapses in living humans is not possible^(16)^.

Different non-invasive procedures for assessing the auditory system are used to understand how CS manifests in humans^(18)^. Until now, a decrease in the amplitude of wave I of the Brainstem Auditory Evoked Potential has been assumed to be one of the main findings indicating the presence of CS in individuals with normal hearing and complaints of speech understanding in noise^(4,7,12,18-20)^. However, other measures have also been widely investigated, such as the potential Frequency Following Response, acoustic stapedial reflex, electrocochleography, and psychoacoustic behavioral tests^(21-24)^.

While specialized literature has made progress in studying CS, caution must be exercised when suggesting its presence, as it is a specific auditory mechanism disorder, and its main manifestation is also observed in other disorders, including the well-established central auditory processing disorder.

Currently, the synaptic rupture between the IHC strand and the primary auditory neurons is called CS and HHL or even “auditory neuropathy.” Some authors use the term HHL, coined by Schaette and McAlpine^(3)^, as a generic term for different auditory disorders that present with normal auditory thresholds. As there is no consensus on the most appropriate terminology and the diagnostic process is still not well defined, diagnosing HHL can be a challenge. One way to contribute to clinical consensus and reduce confusion about idiopathic issues is to identify the terminology adopted in the specialized literature for what is intended to be investigated.

## PURPOSE

To identify pathophysiological definitions used by studies that investigated CS and HHL.

### Search strategy

This study adopted a comprehensive scope review design following the guidelines recommended by the Joanna Briggs Institute Manual for Evidence Synthesis for Scoping Reviews^(25)^ and PRISMA for Scoping Reviews^(26)^.

The research question was elaborated using the acronym PCC: population – studies that proposed to investigate CS; concept – the pathophysiological definition attributed to the term used; context –the subjects of these studies ranged from healthy individuals to those with specific conditions or exposures that were considered pathological. The research methodology involved conducting electrophysiological and/or behavioral tests to gather data. The following question was formulated: what pathophysiological definitions have been adopted by studies that investigated CS and/or HHL in humans?

The database was queried between January and February 2022, and a final search was conducted on February 30th. The selected keywords were extracted from PubMed indexing vocabulary, Medical Subject Headings (MeSH Terms), and the Health Sciences Descriptors Library in English. The descriptors were combined as follows: “Auditory Synaptopathy” or “Neuronal Synaptopathy” or “Hidden Hearing Loss” with “etiology” or “causality” or “diagnosis” (Appendix 1). The EMBASE, PubMed (MEDLINE), CINAHL (EBSCO), and Web of Science databases were searched. The study period was from January 1, 2010, to February 28, 2022, to capture relevant research conducted after the term “Hidden Hearing Loss,” coined in 2011 by Schaette and McAlpine ^((3))^. Studies focusing on humans and employing various study designs, such as observational studies (including case-control, cohort, and cross-sectional studies) and randomized or uncontrolled clinical trials, were included in the analysis. No language restrictions were applied during selection.

### Selection criteria

The selection process was conducted in a blinded and independent manner by two reviewers. Initially, articles were categorized based on their titles and abstracts. Only articles that specifically focused on investigating CS or HHL in human subjects were selected for a more comprehensive evaluation. This evaluation involved carefully reading the articles in full and considering the auditory, behavioral, and/or electrophysiological evaluation procedures used.

### Data analysis

Two authors independently analyzed the articles. To facilitate data comprehension, the collected information was categorized into different topics. These categories included: a) author and year of publication, b) type of study, c) target population, d) adopted terminology, and e) pathophysiological definitions. The data were presented descriptively and the analysis was conducted using a descriptive format.

## RESULTS

**Figure 1:**
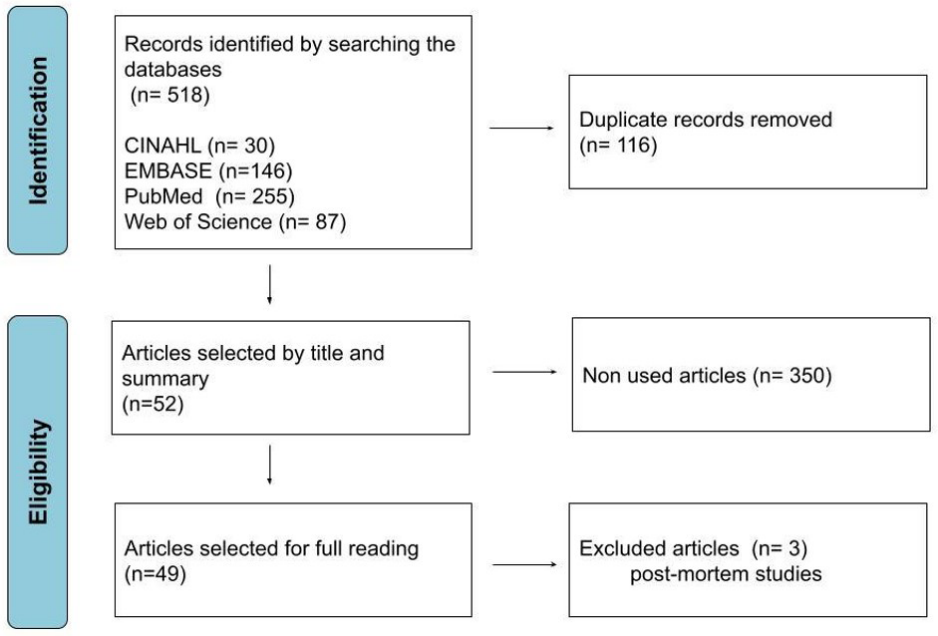
Search and selection flowchart

A total of 518 articles were initially identified through the database search (Figure 1). A total of 116 articles were excluded because of duplicity, leaving 402 articles for screening based on their titles and abstracts. From this screening, 52 articles were selected for full reading. During the second selection phase, three articles were excluded because they focused exclusively on postmortem populations. Consequently, 49 articles were selected for further analysis.

### Characteristics of the studies

Table 1 provides a chronological overview of the key characteristics of the included studies.

**Table 1:** Characterization of the articles included in the review

	**Author (Year)**	**Type of study**	**Terminology adopted population studied**	**Definition**
**1**	Schaette et al.^(3)^ -2011	Control case	● HHL	“Deafferentiation after noise damage predominantly affects high-threshold NA fibers, while a sufficient number of low-threshold fibers remain responsive to sound.”
			● Tinnitus	
**2**	Mehraei et al.^(27)^ -2016	Cross-sectional observational	● CS and HHL - *synonyms*	“Loss of synapses and cochlear nerve terminals that innervate the IHCs.”
			● Healthy	
**3**	Bramhall et al.^(19)^ -2017	Control case	● CS	“Partial loss of IHC synapses of auditory nerve fibers.”
			● Noise exposure	
**4**	Prendergast et al.^(28)^ -2017	Cross-sectional observational	● CS and HHL - *synonyms*	“CS promoted by exposure to noise (often referred to as “HHL”) was demonstrated in a mouse by Kujawa and Liberman (2009).”
			● Noise exposure	
**5**	Grin et al.^(29)^ -2017	Control case	● CS and HHL - *synonyms*	“A synaptopathic lesion that affects the FMTE, which have higher response thresholds and are responsible for encoding higher-intensity sounds.”
			● Noise exposure	
**6**	Paul et al.^(21)^ -2017	Control case	● CS and HHL - *synonyms*	“AN fiber damage that does not alter auditory thresholds.”
			● Tinnitus	
**7**	Wojtczak et al.^(30)^ -2017	Control case	● CS	“Diffuse and permanent loss between IHC and AN synaptic connections after exposure to high-intensity noise, without measurable permanent changes in cochlear function or auditory sensitivity.”
			● Tinnitus	
**8**	Shim et al.^(31)^ -2017	Control case	● CS and HHL - cause/symptom	“”HHL” is characterized as damage to the AS that is not sufficient to produce a threshold shift,the AS partially recovers as thresholds are restored, despite residual physical damage. Selective loss of high-threshold AN or CS fibers may occur without auditory threshold switching due to intact low-threshold fibers.”
			● Tinnitus	
**9**	Paul et al.^(22)^ -2017	Cross-sectional observational	● CS	“Damage of the cochlear synapses necessary for supraliminal abilities, even when the cochlear structures necessary for threshold hearing remain unaffected.”
			● Noise exposure	
**10**	Grose et al.^(4)^ -2017	Control case	● CS	“Suprathreshold deficits in the presence of hearing thresholds within normal limits. In which there is synaptic disruption between the IHC and primary auditory neurons.”
			● Noise exposure	
**11**	Guest et al.^(5)^, -2017	Control case	● CS	“Preferential loss of AN fibers with low spontaneous firing rate and high threshold.”
			● Tinnitus	
**12**	Prendergast et al.^(32)^ -2017	Cross-sectional observational	● CS	“Loss of synapses between IHC and AN fibers.”
			● Noise exposure	
**13**	Valderrama et al.^(33)^ -2018	Cross-sectional observational	● CS and HHL - *synonyms*	“Theory known as “HHL”, in which CS in humans is the hypothesis to explain speech intelligibility deficits in the presence of normal audiogram.”
			● Noise exposure	
**14**	Guest et al.^(34)^ -2018	Cross-sectional observational	● CS	“Loss of synapses between the IHC and the AN fibers, which can occur without cell loss or permanent threshold elevation.”
			● Speech comprehension difficulty	
**15**	Bramhall et al.^(6)^ -2018	Control case	● CS	“Selective damage to the afferent auditory nerve synapses in the IHC, with auditory thresholds within normal limits.”
			● Noise exposure	
**16**	Guest et al.^(35)^ -2019	Cross-sectional observational	● CS	“Loss of synapses between IHC and AN fibers.”
			● Tinnitus	
**17**	Ridley et al.^(36)^ -2019	Control case	● CS and HHL - cause/symptom	“Damage AN's FBTE and FMTE, which are involved in processing moderate to loud sounds, and are more resistant to masking by background noise. A possible cause for HHL is CS.”
			● SNHL	
**18**	Grose et al.^(12)^ -2019	Cross-sectional observational	● CS	“Permanent damage to the synapses between the IHC and the AN fibers, insufficient to result in a permanent elevation of auditory thresholds.”
			● Aging	
**19**	Bhatt & Wang^(37)^ -2019	Control case	● CS	“Irreversible damage to the synaptic connections between the IHC and AN. This noise-induced CS cannot be detected by assessing hearing thresholds because noise exposure does not always cause IHC or EHC loss.”
			● Noise exposure	
**20**	Johannesen et al.^(38)^ -2019	Cross-sectional observational	● CS	“Animal studies have shown that the number of AN fibers decreases with increasing age in healthy cochleae. Some authors speculate that CS and/or deafferentation may be responsible for difficulties in understanding speech in the elderly.”
			● Healthy	
**21**	Risato-Lago et al.^(39)^ -2019	Control case	● HHL	“Condition in which AS damage does not produce threshold change or there is partial recovery as thresholds are restored to original levels despite residual physical damage.”
			● Sickle cell anemia	
**22**	Guest et al.^(18)^ -2019	Control case	● CS	“Loss of synapses between cochlear IHC and AN fibers, without generalized loss of hair cells.”
			● Healthy	
**23**	Prendergast et al.^(40)^ -2019	Cross-sectional observational	● CS	“Kujawa and Liberman^(1)^ described the phenomenon now known as CS (…) loss of synapses with unchanged absolute thresholds, but associated with a reduction in Wave I..”
			● Noise exposure	
**24**	Megha et al.^(41)^ -2019	Control case	● CS and HHL - *synonyms*	“Temporary change in the threshold, with damage to the connections between the fibers of the AN and the IHC of the cochlea, causing CS. This type of damage to the synapse, which does not permanently raise the threshold, is called HHL.”
			● Noise exposure	
**25**	Keshishzadeh et al.^(42)^ -2020	Control case	● CS	“Irreversible loss of AN synapses and degeneration of cochlear neurons, without damage to cochlear sensory hair cells.”
			● Speech comprehension difficulty	
**26**	Mepani et al.^(43)^ -2020	Cross-sectional observational	● CS	“It is “hidden” because neural degeneration per se does not raise behavioral thresholds or electrophysiological thresholds until it becomes extreme.”
			● Healthy	
**27**	Couth et al.^(44)^ -2020	Control case	● CS and HHL - *synonyms*	“Loss of synapses between IHC and spiral ganglion neurons.”
			● Noise exposure	
**28**	Parker et al.^(10)^ -2020	Cross-sectional observational	● CS and HHL - cause/symptom	“Loss of the synaptic connection between the IHC and the AN fibers, impairing the ability to understand in adverse listening situations.”
			● Healthy	
**29**	Grant et al.^(9)^ -2020	Cross-sectional observational	● CS	“Damage of synapses between cochlear and IHC nerve fibers even as hair cells and thresholds recover.”
			● Healthy	
**30**	Kara et al.^(45)^ -2020	Control case	● CS and HHL - *synonyms*	“Loss of IHC synapses without any evidence of increased auditory thresholds.”
			● Tinnitus	
**31**	Bramhall et al.^(8)^ -2020	Control case	● CS	“Loss of synaptic connections between the IHC and their auditory afferent nerve fiber targets.”
			● Tinnitus and noise exposure	
**32**	Shehorn et al.^(46)^ -2020	Control case	● CS	“Loss of connections between the IHC and the auditory nerve fibers, in the absence of permanent threshold change.”
			● Speech comprehension difficulty	
**33**	Okada et al.^(47)^, -2020	Cross-sectional observational	● CS	“Reduced cochlear efferent innervation and a loss of afferent synapses between the AN and sensory cells.”
			● CHL	
**34**	Washnik et al.^(48)^ -2020	Control case	● CS and HHL - *synonyms*	“Irreversible damage to the synaptic connections between the cochlear IHC and the NA fibers. This type of peripheral hearing loss can lead to impaired speech perception and has been called “HHL”.
			● Noise exposure	
**35**	Carcagno & Plack^(49)^ -2020	Cross-sectional observational	● CS	“Permanent loss of synapses between IHC and AN fibers.”
			● Aging	
**36**	Marmel et al.^(50)^ -2020	Control case	● CS and HHL - *synonyms*	“Subclinical auditory pathology that could explain some hearing difficulties observed despite (almost) normal audiometric thresholds.”
			● Tinnitus	
**37**	Carcagno & Plack^(51)^ -2021	Cross-sectional observational	● CS	“Permanent loss of synapses between IHC and AN fibers.”
			● Aging	
**38**	Shim et al.^(52)^ -2021	Control case	● CS	“The selective loss of high-threshold fibers and/or high-threshold synaptopathy.”
			● Unilateral tinnitus	
**39**	Bal et al.^(11)^ -2021	Control case	● CS and HHL - *synonyms*	“Damage to cochlear nerve fibers, especially in the FBTE, with disruption of synaptic communication between the sensory IHC and cochlear nerve fiber subsets.”
			● Noise exposure	
**40**	Suresh et al.^(20)^ -2021	Control case	● CS and HHL - cause/symptom	“Reduction in the number of synaptic strands between the IHC and the AN fibers without affecting the audiometric thresholds.”
			● Noise exposure	
**41**	Nam et al.^(53)^ -2021	Control case	● CS and HHL - *synonyms*	“When synapses are damaged, nerve fibers subsequently degenerate.”
			● Noise exposure	
**42**	Megha et al.^(13)^ -2021	Control case	● SC and HHL - *synonyms*	“Loss of synapses and cochlear nerve endings that innervate the IHC.”
			● Noise exposure and aging	
**43**	Wang et al.^(7)^ -2021	Cross-sectional observational	● CS	“Permanent dysfunction at the junctions between the IHC and the AN fibers caused by low-grade trauma to the inner ear, typically associated with noise exposure, insufficient for permanent elevation of thresholds.”
			● Noise exposure	
**44**	Vasilkov et al.^(54)^ -2021	Control case	● CS	“Degeneration of the synaptic terminals of spiral ganglion cells, which precedes IHC damage in the aging process.”
			● Aging	
**45**	Bramhall et al.^(23)^ -2021	Control case	● CS	“Loss of connection between the IHI and their afferent AN fiber targets.”
			● Noise exposure	
**46**	Chen et al.^(55)^ -2021	Cross-sectional observational	● CS	“Affects the connection between the IHCs, with dysfunction in the FBTE, reducing the ability to perceive speech in a noisy environment.”
			● Aging	
**47**	Edvall et al.^(56)^ -2022	Control case	● CS	“Loss of synaptic connection between the IHC and the afferent fibers of the AN.”
			● Tinnitus	
**48**	Turner et al.^(57)^ -2022	Control case	● CS	“Synaptic change between the IHC and the auditory nerve fibers.”
			● Tinnitus	
**49**	Bramhall et al.^(24)^ -2022	Control case	● CS	“Loss of synapses between the IHC and afferent auditory nerve fibers.”
			● Noise exposure	

**Caption:** CS = Cochlear synaptopathy; HHL = Hidden Hearing Loss; AN = Auditory Nerve; IHC = Inner Hair Cell; FMTE = Medium Rate Spontaneous Fibers; FBTE = Low Rate Spontaneous Fibers; AS = Auditory System; EHC = External Hair Cell; . SNHL = Sensorineural Hearing Loss; CHL = Conductive Hearing Loss

In terms of study design, the analysis revealed that the majority of the studies 31 (63.2%) were case-control studies, while 36.7% (18 of 49) were cross-sectional observational studies.

### Terminology used and its application

Out of the 49 articles included in the study, a majority of them, 61.2% (30/49), utilized the terminology “CS” to refer to the specific phenomenon that has been researched. A smaller proportion of articles, 4.1% (2/49), solely employed the term “HHL.” A significant proportion of the selected articles (34.7%, 17/49) adopted both terms.

Of the articles that adopted CS terminology (Studies 3, 7, 10, 11, 12, 14, 15, 16, 18, 19, 20, 21, 22, 23, 25, 26, 29, 31, 32, 33, 35, 37, 43, 44, 45, 46, 47, 48, and 49), the most commonly used definitions were those of Kujawa and Liberman^(1)^, Makary et al.^(58)^, Sergeyenko et al.^(59)^, and Liberman and Kujawa^(60)^. According to the definitions adopted in these articles, CS is the loss of synapses between the IHC and fibers of the auditory nerve, which produces lesions in fibers with a low rate of spontaneous discharge and a high threshold in the absence of permanent alteration of the auditory threshold^(1)^. A reduction in cochlear efferent innervation and loss of afferent synapses between the cochlear nerve and sensory cells has also been reported^(59,60)^. These authors agree that CS has been demonstrated in studies on animals, rodents, and primates, mainly as a consequence of exposure to high levels of sound intensity^(1,27-29)^. Due to the impairment of synapses with efferent fibers, there is an impairment in acoustic stimulus encoding, since it initiates auditory input in the central auditory system. Therefore, it can be inferred that studies using CS terminology aimed to study a phenomenon restricted to a specific location of injury or auditory mechanisms. Furthermore, there are no differences between authors regarding its definition^(1,58-60)^.

The term “HHL” was used in two studies (Studies 1 and 21). Among the articles that adopted it to designate the objective of the study, one described it as a condition in which there is an alteration in the auditory system without any change in the auditory threshold (Study 21). Thus, the authors considered HHL to be a sign of hearing disease and not the cause itself. In another article (Study 1), HHL was described as deafferentiation between the cochlear nerve fibers and the SG, in which the auditory thresholds remained within normal limits and there was impaired function of efferent fibers that projected from the brainstem to the cochlea.

The terms CS and HHL were adopted together in studies 2, 4, 5, 6, 8, 13, 17, 24, 27, 28, 30, 34, 36, 39, 40, 41, and 42. Of the 17 articles, 76.5% (13/17) assumed HHL to be a synonym for CS (Studies 2, 4, 5, 6, 13, 24, 27, 30, 34, 36, 39, 41, and 42) using the definitions by Schaette et al.^(3)^, Kuwaja and Liberman^(1)^. Meanwhile, 23.5% (04/17) differentiated them on the pathological phenomenon and its signs (Studies 8, 17, 28, and 40). In other words, the authors assumed that CS was a possible cause of HHL.

The term HHL is used as a generic designation for at least 14 diseases that affect the auditory system and cause complaints of speech understanding in the absence of peripheral hearing loss. This term is widely accepted for different pathophysiological descriptions, as long as there are no changes in the auditory threshold. Therefore, caution is required when associating it as a synonym for CS, which is characterized in a very specific manner, especially regarding its pathophysiological processes.

It is still necessary to add that, during the search for articles, in two of the selected articles, the use of the term “auditory neuropathy spectrum disorder” was observed, referring to cochlear deafferentiation. Auditory neuropathy is a well-established condition in which cranial nerve VIII is compromised due to changes in neural synchrony during synaptic transmission. The location of the auditory nerve involvement is variable, and there may be peripheral hearing loss of different degrees, unilateral or bilateral, symmetrical, or asymmetrical. Therefore, auditory neuropathy differs from CS^(61)^.

### Study Populations

The selected studies included different populations and conditions. The population exposed to high sound pressure levels was the research objective of 44.8% (22/49) of the studies, followed by the population with uni- or bi-lateral tinnitus in 24.4% (12/49), 12.2% (06/49), respectively in the aging condition and 10.2% (05/49) in the “healthy” condition. Conductive hearing loss, sensorineural hearing loss, and sickle cell anemia accounted for 2.0% (01/49) of the studies. Three articles studied more than one condition, two addressed exposure to high levels of sound pressure and tinnitus, and one studied exposure to high levels of sound pressure and aging.

Most of the conditions addressed by these studies were identified as risk factors for CS (e.g. exposure to noise, aging, and tinnitus). Exposure to high sound pressure levels has been the most studied (Studies 3, 4, 5, 6, 10, 12, 13, 15, 16, 19, 23, 24, 27, 31, 34, 39, 40, 41, 42, 43, 45, and 49), possibly because it presents mechanisms of physiological damage that are known and relatively amenable to control CS. However, it is the most likely etiological factor in this pathology. The second most investigated condition was tinnitus (Studies 1, 7, 8, 9, 11, 13, 30, 31, 36, 38, 47, and 48), which is also an indication of CS. However, some considerations regarding this condition are necessary, as it is a heterogeneous symptom in etiology, location, acoustic characteristics, and associated comorbidities^(62)^. Tinnitus is often associated with hearing loss, acoustic trauma, exposure to high levels of sound pressure, use of ototoxic drugs, cardiovascular alterations, temporomandibular disorders, or the absence of apparent causes^(31)^. Thus, to infer that tinnitus was caused by CS, other factors must be excluded. The tinnitus studies included in this review did not mention excluding or documenting the presence of other conditions in their samples, except for hearing loss. The same occurs for aging (Studies 18, 35, 37, 42, 44, and 46), “healthy” conditions (Studies 2, 20, 22, 26, 28, and 29), and speech comprehension complaints (14, 25, 32). To confirm the presence of CS in these populations, it is necessary to exclude changes in the central nervous system, because they also promote changes in suprathreshold abilities^(63)^.

Conditions of conductive hearing loss (Study 33), sensorineural hearing loss (Study 17), and sickle cell anemia (Study 21) were also found in the present study. A study that investigated individuals with conductive hearing loss used the term CS. However, this study indicates that chronic conductive hearing loss in adults may be a risk factor for the development of CS. The study on sensorineural hearing loss used the terms CS and HHL as synonyms and was applicable to the studied conditions. However, there was no alteration in the auditory threshold of synaptopathy^(1)^; thus, the designation of what was being investigated may have been mistaken. Finally, the study of individuals with sickle cell anemia used the term HHL only generically to indicate alterations in the auditory system that did not affect the audiogram results.

## CONCLUSION

CS terminology was the most commonly used by the included studies, all of which referred to the pathophysiological process of deafferentiation between the cochlear nerve fibers and IHC. Most studies that adopted both terms used them synonymously, whereas others described HHL as a possible consequence of CS. A smaller proportion of the studies solely used the term HHL, considering it as an indication of hearing impairment.

## Appendix 1 Search strategy used according to the database

**Table:**

**Data base**	Search Strategy
**PUBMED**	(*(“Auditory Synaptopathy”[All Fields] OR ((“neuron s”[All Fields] OR “neuronal”[All Fields] OR “neuronally”[All Fields] OR “neuronals”[All Fields] OR “neurone s”[All Fields] OR “neurones”[All Fields] OR “neuronic”[All Fields] OR “neurons”[MeSH Terms] OR “neurons”[All Fields] OR “neuron”[All Fields] OR “neurone”[All Fields]) AND (“synaptopathies”[All Fields] OR “synaptopathy”[All Fields])) OR “Hidden Hearing Loss”[All Fields]) AND “etiology”[All Fields]) OR “causality”[All Fields] OR “diagnosis”[All Fields].*
**EMBASE**	('auditory synaptopathy'/exp OR 'auditory synaptopathy' OR 'neuronal synaptopathy' OR 'hidden hearing loss'/exp OR 'hidden hearing loss') AND ('etiology'/exp OR 'etiology') OR 'causality'/exp OR 'causality' OR 'diagnosis'/exp OR 'diagnosis'
**CINAHL**	*Auditory Synaptopathy” OR “Neuronal Synaptopathy” OR “Hidden Hearing Loss” AND etiology OR causality OR diagnosis.*
**Web of Science**	*“Auditory Synaptopathy” (All Fields) OR “Neuronal Synaptopathy” (All Fields) OR “Hidden Hearing Loss” (All Fields) AND etiology (All Fields) OR causality (All Fields) OR diagnosis (All Fields).*
