# Supplementary material for: Cochlear synaptopathy and hidden hearing loss: a scoping review
Source: Codas. 2023 Nov 20;36(2):e20230032. doi: 10.1590/2317-1782/20232023032en (PMC10715634; doi:10.1590/2317-1782/20232023032en)
Supplement: Translation

# Translation: Sinaptopatia coclear e perda auditiva oculta: uma revisão de escopo

**DOI:** 10.1590/2317-1782/20232023032pt

**Authors:** Marina de Figueiredo Colla, Pamela Papile Lunardelo, Fernanda Abalen Martins Dias

**Affiliations:** 1 Departamento de Fonoaudiologia, Pontifícia Universidade Católica de Minas Gerais – PUC MG - Belo Horizonte (MG), Brasil.; 2 Programa de Pós-graduação em Psicobiologia, Universidade de São Paulo de Ribeirão Preto – USP RP - Ribeirão Preto (SP), Brasil.

**Keywords:** Audição, Perda Auditiva Oculta, Sinaptopatia Coclear, Terminologia

## Abstract

**Objetivo:**

Identificar as definições fisiopatológicas adotadas pelos estudos que investigaram a “sinaptopatia coclear” (SC) e “perda auditiva oculta” (PAO).

**Estratégia de pesquisa:**

Utilizou-se a combinação de unitermos *“Auditory Synaptopathy” or “Neuronal Synaptopathy” or “Hidden Hearing Loss” com “etiology” or “causality” or “diagnosis”* nas bases de dados EMBASE, Pubmed (MEDLINE), CINAHL (EBSCO) e Web of Science.

**Critérios de seleção:**

Incluiu-se estudos que investigaram a SC ou PAO em humanos com procedimentos comportamentais e/ou eletrofisiológicos.

**Análise dos dados:**

Realizou-se a análise e extração de dados quanto a terminologia, definição e população estudada.

**Resultados:**

Foram incluídos 49 artigos. Destes, 61,2% utilizaram a terminologia SC, 34,7% ambos os termos e 4,1% utilizaram PAO. As condições mais estudadas foram exposição ao ruído e zumbido.

**Conclusão:**

A terminologia SC foi empregada na maioria dos estudos, com referência ao processo fisiopatológico de desaferenciação entre as fibras do nervo coclear e as células ciliadas internas

## INTRODUÇÃO

A sinaptopatia coclear (SC) é caracterizada pela desaferenciação entre as fibras do nervo coclear e as células ciliadas internas (CCI) no gânglio espiral (GE). Os neurônios cocleares e suas vulneráveis conexões sinápticas são os principais alvos de alguns agentes patológicos, com acometimento predominante de fibras de baixa taxa espontânea e alto limiar^(1)^. Este processo patológico é extra-axial e precede a mudança permanente do limiar auditivo, desta forma, a deterioração sináptica ocorre mesmo quando as CCI permanecem íntegras^(2,3)^.

Ao longo dos anos, diferentes nomenclaturas foram atribuídas ao perfil auditivo caracterizado pela presença de audição normal e déficits supraliminares^(4)^, isto porque, esta manifestação pode estar associada a diferentes doenças que acometem o sistema auditivo. Atualmente, as designações mais comuns atribuídas a desaferenciação supracitada são SC ou perda auditiva oculta (PAO).

As principais manifestações clínicas da SC são a dificuldade de compreensão de fala no ruído, zumbido e hiperacusia na presença de audição normal^(3,5-11)^. Entretanto, deve-se ressaltar que tais manifestações são comuns a variados processos patológicos auditivos e/ou otológicos e não apenas à SC.

Diferentes fatores são apontados como causa da SC, sendo a exposição ao ruído e o envelhecimento os principais deles^(1,3,4,8,12,13)^. A exposição a níveis de pressão sonora elevados pode gerar danos entre as sinapses das CCI e as terminações nervosas do nervo auditivo^(1,14)^, pois ocasiona liberação excessiva de glutamato no receptor pós-sináptico do nervo coclear, promovendo excitotoxicidade e inchaço nas fibras terminais do GE^(1,15)^, iniciando uma cascata degenerativa, marcada pela elevação temporária do limiar auditivo, considerada transitória^(1)^.

Outro possível fator causal é o envelhecimento. A partir da análise do osso temporal em estudos *post-mortem* ^(16)^ identifica-se a perda de fibras do nervo auditivo mesmo na ausência de morte das CCI, ou ainda, perda mais acentuada destas fibras quando já existe morte celular. As mudanças no limiar auditivo ocorrem apenas quando a perda neuronal excede cerca de 80% a 90%^(2,17)^. Embora este seja o procedimento “padrão ouro” para indicar a SC, a quantificação de sinapses cocleares em humanos vivos não é uma medida possível^(16)^.

Diferentes procedimentos não invasivos de avaliação do sistema auditivo são utilizados para compreender como a SC se manifesta em humanos^(18)^. Até o presente momento, assume-se a diminuição da amplitude da onda I do Potencial Evocado Auditivo de Tronco Encefálico (PEATE) como um dos principais achados que poderia indicar a presença de SC em indivíduos com audição normal e queixa de compreensão de fala no ruído^(4,7,12,18-20)^. Entretanto, outras medidas também são amplamente investigadas, como o potencial *Frequency Following Response*, o reflexo acústico do músculo da orelha média, a eletrococleografia e alguns testes comportamentais psicoacústicos^(21-24)^.

Ainda que a literatura especializada tenha avançado nos estudos sobre a SC, é necessário ter cautela ao indicar a possibilidade de sua presença, pois se trata de uma lesão em um mecanismo auditivo muito específico e a sua principal manifestação, também está presente em outros transtornos, o que inclui o já bem estabelecido transtorno do processamento auditivo central.

Atualmente, a ruptura sináptica entre a fita das CCI e os neurônios auditivos primários é denominada de SC e PAO ou até mesmo de “neuropatia auditiva”. Alguns autores apontam o termo PAO, cunhado por Schaette e McAlpine^(3)^, como genérico para se referir a diferentes disfunções auditivas com presença de limiares auditivos dentro da normalidade. A falta de consenso quanto ao termo mais adequado pode dificultar o processo de diagnóstico desta patologia que ainda não está bem definida, tornando-se uma problemática. Uma das formas de contribuir para o consenso clínico e reduzir a confusão entre questões idiopáticas, é identificar qual a terminologia adotada na literatura especializada para o que se pretende investigar.

## OBJETIVO

Identificar as definições fisiopatológicas adotadas pelos estudos que investigaram a SC e a PAO.

### Estratégia de pesquisa

O presente estudo baseou-se no delineamento de revisão de escopo a partir das recomendações da *Joanna Briggs Institute Manual for Evidence Synthesis for Scoping Reviews* ^(25)^ e do *PRISMA for Scoping Reviews* ^(26)^.

A pergunta de pesquisa foi elaborada utilizando-se a sigla PCC: POPULAÇÃO - estudos que se propuseram a investigar a SC e/ou a PAO; Conceito – a definição fisiopatológica atribuída ao termo utilizado; Contexto – estudos com humanos, hígidos ou com alguma condição ou exposição considerada patológica, com testes eletrofisiológicos e/ou comportamentais. Formulou-se a pergunta: Quais definições fisiopatológicas adotadas pelos estudos que propuseram a investigação da SC e/ou da PAO em humanos?

O período de busca nas bases de dados correspondeu aos meses de janeiro e fevereiro de 2022, sendo a data da última busca o dia 30 de fevereiro. Os unitermos selecionados foram extraídos do vocabulário de indexação da PubMed, *Medical Subject Headings* (MeSH Terms) e na biblioteca de Descritores em Ciência da Saúde (DeCS), no idioma inglês. Os descritores foram combinados da seguinte forma: *“Auditory Synaptopathy” or “Neuronal Synaptopathy” or “Hidden Hearing Loss” com “etiology” or “causality” or “diagnosis”* (Apêndice 1). As bases de dados pesquisadas foram a EMBASE, Pubmed (MEDLINE), CINAHL (EBSCO) e Web of Science. Estipulou-se como filtro o período de 01 de janeiro de 2010 até 30 de fevereiro de 2022 devido ao termo “*Hidden Hearing Loss”* ter sido cunhado apenas em 2011 por Schaette e McAlpine^(3)^. Ainda, selecionou-se estudos em humanos e o tipo do estudo (estudos observacionais – caso-controle, coortes, transversais – e ensaio clínico randomizado ou não controlado), sem restrições de idioma.

### Critérios de seleção

A seleção dos estudos foi realizada de forma cega e independente, por dois revisores. Os artigos foram triados a partir de seu título e resumo, sendo selecionados para leitura na íntegra aqueles que objetivaram investigar a SC ou PAO em humanos por meio de procedimentos de avaliação do sistema auditivo, comportamentais e/ou eletrofisiológicos.

### Análise dos dados

A análise dos artigos foi conduzida de forma independente por dois autores. As informações coletadas foram divididas em diferentes tópicos, a fim de possibilitar a compreensão dos dados coletados: a) autor e ano da publicação; b) tipo de estudo; c) população alvo; d) terminologia adotada; e) definição fisiopatológica.

Os dados estão apresentados de forma descritiva e a análise realizada no formato descritivo.

## RESULTADOS

A partir da busca nas bases de dados foram identificados inicialmente 518 artigos como apresentados na Figura 1, dos quais 116 foram excluídos por duplicidade. Foram triados pelo título e resumo 402 artigos e selecionados para leitura na íntegra 52. Durante a segunda fase de seleção, três artigos foram excluídos por incluírem apenas a população *post-mortem*, com isto, foram selecionados para análise 49 artigos.

**Figura 1:** Fluxograma de busca e seleção

### Características dos estudos

A Tabela 1 apresenta as características dos estudos incluídos, em ordem cronológica de publicação.

**Tabela 1:** Caracterização dos artigos incluídos na revisão

	**Autor (Ano)**	**Tipo do estudo**	**Terminologia adotada**	**Definição**
			**população estudada**	
**1**	Schaette et al.^(3)^ -2011	Caso controle	● PAO	“Desaferenciação após dano por ruído, afetando predominantemente as fibras do NA de alto limiar, enquanto um número suficiente de fibras de baixo limiar permanece responsivo ao som.”
			● Zumbido	
**2**	Mehraei et al.^(27)^ -2016	Observacional transversal	● SC e PAO - *sinônimos*	“Perda das sinapses e de terminais do nervo coclear que inervam as CCI.”
			● Hígidos	
**3**	Bramhall et al.^(19)^ -2017	Caso controle	● SC	“Perda parcial das sinapses das CCI das fibras nervosas auditivas.”
			● Exposição ao ruído	
**4**	Prendergast et al.^(28)^ -2017	Observacional transversal	● SC e PAO - *sinônimos*	“A SC promovida pela exposição ao ruído (muitas vezes referida como “PAO”) foi demonstrada em um camundongo por Kujawa e Liberman (2009).”
			● Exposição ao ruído	
**5**	Grin et al.^(29)^ -2017	Caso controle	● SC e PAO - *sinônimos*	“Lesão sinaptopática que acomete as FMTE, que têm limiares de resposta mais altos e são responsáveis por codificar sons de maior intensidade.”
			● Exposição ao ruído	
**6**	Paul et al.^(21)^ -2017	Caso controle	● SC e PAO - *sinônimos*	“Danos nas fibras do NA que não alteram os limiares auditivos.”
			● Zumbido	
**7**	Wojtczak et al.^(30)^ -2017	Caso controle	● SC	“Perda difusa e permanente entre as conexões sinápticas das CCI e do NA após a exposição ao ruído de forte intensidade, sem alterações permanentes mensuráveis na função coclear ou sensibilidade auditiva.”
			● Zumbido	
**8**	Shim et al.^(31)^ -2017	Caso controle	● SC e PAO - *causa/sintoma*	“A “PAO” caracteriza-se como um dano ao SA que não é suficiente para produzir uma mudança de limiar, o SA se recupera parcialmente a medida em que os limiares são restaurados, apesar do dano físico residual. A perda seletiva de fibras do NA de alto limiar ou SC pode ocorrer sem alteração do limiar auditivo devido às fibras de baixo limiar intactas.”
			● Zumbido	
**9**	Paul et al.^(22)^ -2017	Observacional transversal	● SC	“Dano das sinapses cocleares necessárias para as habilidades supraliminares, mesmo quando as estruturas cocleares necessárias para a detecção auditiva permanecem inalteradas.”
			● Exposição ao ruído	
**10**	Grose et al.^(4)^ -2017	Caso controle	● SC	“Déficits supralimiares na presença limiares auditivos dentro dos padrões de normalidade. Em que existe a ruptura sináptica entre as CCI e os neurônios auditivos primários.”
			● Exposição ao ruído	
**11**	Guest et al.^(5)^, -2017	Caso controle	● SC	“Perda preferencial de fibras do NA com baixa taxa de disparo espontâneo e alto limiar.”
			● Zumbido	
**12**	Prendergast et al.^(32)^ -2017	Observacional transversal	● SC	“Perda das sinapses entre as CCI e as fibras do NA.”
			● Exposição ao ruído	
**13**	Valderrama et al.^(33)^ -2018	Observacional transversal	● SC e PAO - *sinônimos*	“Teoria conhecida como “PAO”, em que a SC em humanos é a hipótese para explicar déficits de inteligibilidade de fala na presença de audiograma normal.”
			● Exposição ao ruído	
**14**	Guest et al.^(34)^ -2018	Observacional transversal	● SC	“Perda de sinapses entre as CCI e as fibras do NA, que pode ocorrer sem perda de células ou elevação permanente do limiar.”
			● Dificuldade de compreensão de fala	
**15**	Bramhall et al.^(6)^ -2018	Caso controle	● SC	“Dano seletivo das sinapses do nervo auditivo aferente nas CCI, com limiares auditivos dentro dos padrões de normalidade.”
			● Exposição ao ruído	
**16**	Guest et al.^(35)^ -2019	Observacional transversal	● SC	“Perda de sinapses entre as CCI e as fibras do NA.”
			● Zumbido	
**17**	Ridley et al.^(36)^ -2019	Caso controle	● SC e PAO - *causa/sintoma*	“Dano nas FBTE e FMTE do NA, que estão envolvidos no processamento de sons de intensidade moderada a forte e são mais resistentes ao mascaramento por ruído de fundo. Uma possível causa para a PAO é a SC.”
			● PASN	
**18**	Grose et al.^(12)^ -2019	Observacional transversal	● SC	“Dano permanente nas sinapses entre as CCI e as fibras do NA, insuficiente para resultar em uma elevação permanente dos limiares auditivos.”
			● Envelhecimento	
**19**	Bhatt and Wang^(37)^ -2019	Caso controle	● SC	“Danos irreversíveis nas conexões sinápticas entre as CCI e NA. Esta SC induzida por ruído não pode ser detectada avaliando-se limiares auditivos porque a exposição ao ruído nem sempre causa perda de CCI ou CCE.”
			● Exposição ao ruído	
**20**	Johannesen et al.^(38)^ -2019	Observacional transversal	● SC	“Estudos em animais mostraram que o número de fibras do NA diminui com o aumento da idade de cócleas saudáveis. Alguns autores especulam que a SC e/ou a desaferenciação podem ser responsáveis por dificuldades na compreensão de fala em idosos.”
			● Hígidos	
**21**	Risato-Lago et al.^(39)^ -2019	Caso controle	● PAO	“Condição na qual o dano do SA não produz mudança de limiar ou há recuperação parcial a medida em que os limites são restaurados aos níveis originais, apesar do dano físico residual.”
			● Anemia Falciforme	
**22**	Guest et al.^(18)^ -2019	Caso controle	● SC	“Perda de sinapses entre as CCI da cóclea e fibras do NA, sem perda generalizada de células ciliadas.”
			● Hígidos	
**23**	Prendergast et al.^(40)^ -2019	Observacional transversal	● SC	“Kujawa e Liberman (2009)^(1)^ descreveram o fenômeno hoje conhecido como SC (…)perda de sinapses com limiares absolutos inalterados, mas associada à redução da Onda I.”
			● Exposição ao ruído	
**24**	Megha et al.^(41)^ -2019	Caso controle	● SC e PAO - *sinônimos*	“Mudança temporária do limiar, com dano nas conexões entre as fibras do NA e as CCI da cóclea, causando SC. Esse tipo de dano à sinapse, que não causa elevação permanente do limiar, é denominado PAO.”
			● Exposição ao ruído	
**25**	Keshishzadeh et al.^(42)^ -2020	Caso controle	● SC	“Perda irreversível de sinapses do NA e degeneração de neurônios cocleares, sem lesão das células ciliadas sensoriais cocleares.”
			● Dificuldade de compreensão de fala	
**26**	Mepani et al.^(43)^ -2020	Observacional transversal	● SC	“É “oculta” porque a degeneração neural não eleva os limiares comportamentais e eletrofisiológicos até se tornar extrema.”
			● Hígidos	
**27**	Couth et al.^(44)^ -2020	Caso controle	● SC e PAO - *sinônimos*	“Perda de sinapses entre as CCI e os neurônios do gânglio espiral.”
			● Exposição ao ruído	
**28**	Parker et al.^(10)^ -2020	Observacional transversal	● SC e PAO - *causa/sintoma*	“Perda da conexão sináptica entre as CCI e as fibras do NA, prejudicando a capacidade de compreensão em situações adversas de escuta.”
			● Hígidos	
**29**	Grant et al.^(9)^ -2020	Observacional transversal	● SC	“Dano das sinapses entre fibras nervosas cocleares e as e CCI, mesmo quando as células ciliadas e os limiares se recuperam.”
			● Hígidos	
**30**	Kara et al.^(45)^ -2020	Caso controle	● SC e PAO - *sinônimos*	“Perda das sinapses das CCI sem qualquer evidência de aumento dos limiares auditivos.”
			● Zumbido	
**31**	Bramhall et al.^(8)^ -2020	Caso controle	● SC	“Perda das conexões sinápticas entre as CCI e seus alvos de fibras nervosas auditivas aferentes.”
			● Zumbido e exposição ao ruído	
**32**	Shehorn et al.^(46)^ -2020	Caso controle	● SC	“Perda de conexões entre as CCI e as fibras nervosas auditivas, na ausência de mudança permanente do limiar.“
			● Dificuldade de compreensão de fala	
**33**	Okada et al.^(47)^, -2020	Observacional transversal	● SC	“Redução na inervação eferente coclear e uma perda de sinapses aferentes entre o NA e as células sensoriais.”
			● PAC	
**34**	Washnik et al.^(48)^ -2020	Caso controle	● SC e PAO - *sinônimos*	“Danos irreversíveis às conexões sinápticas entre as CCI da cóclea e as fibras do NA. Esse tipo de perda auditiva periférica pode levar à percepção prejudicada da fala e tem sido chamada de “PAO” .”
			● Exposição ao ruído	
**35**	Carcagno & Plack^(49)^ -2020	Observacional transversal	● SC	“Perda permanente de sinapses entre as CCI e as fibras do NA.”
			● Envelhecimento	
**36**	Marmel et al.^(50)^ -2020	Caso controle	● SC e PAO - *sinônimos*	“Patologia auditiva subclínica que poderia explicar algumas dificuldades auditivas observadas apesar dos limiares audiométricos (quase) normais.”
			● Zumbido	
**37**	Carcagno & Plack^(51)^ -2021	Observacional transversal	● SC	“Perda permanente de sinapses entre as CCI e as fibras do NA.”
			● Envelhecimento	
**38**	Shim et al.^(52)^ -2021	Caso controle	● SC	“A perda seletiva de fibras de alto limiar e/ou sinaptopatia de alto limiar.”
			● Zumbido unilateral	
**39**	Bal et al.^(11)^ -2021	Caso controle	● SC e PAO - *sinônimos*	“Dano às fibras do nervo coclear, especialmente nas FBTE, com interrupção da comunicação sináptica entre as CCI sensoriais e subconjuntos de fibras do nervo coclear.”
			● Exposição ao ruído	
**40**	Suresh et al.^(20)^ -2021	Caso controle	● SC e PAO - *causa/sintoma*	“Redução do número de fitas sinápticas entre as CCI e as fibras do NA sem afetar os limiares audiométricos.”
			● Exposição ao ruído	
**41**	Nam et al.^(53)^ -2021	Caso controle	● SC e PAO - *sinônimos*	“Quando as sinapses são danificadas, as fibras nervosas posteriormente se degeneram.”
			● Exposição ao ruído	
**42**	Megha et al.^(13)^ -2021	Caso controle	● SC e PAO - *sinônimos*	“Perda de sinapses e terminais nervosos cocleares que inervam as CCI.”
			● Exposição ao ruído e envelhecimento	
**43**	Wang et al.^(7)^ -2021	Observacional transversal	● SC	“Disfunção permanente nas junções entre as CCI e as fibras do NA causada por trauma de baixo grau na orelha interna, tipicamente associado à exposição ao ruído, insuficiente para elevação permanente dos limiares.”
			● Exposição ao ruído	
**44**	Vasilkov et al.^(54)^ -2021	Caso controle	● SC	“Degeneração dos terminais sinápticos das células ganglionares espirais, que precede o dano das CCI no processo de envelhecimento.”
			● Envelhecimento	
**45**	Bramhall et al.^(23)^ -2021	Caso controle	● SC	“Perda da conexão entre as CCI e seus aferentes alvos de fibra do NA.”
			● Exposição ao ruído	
**46**	Chen et al.^(55)^ -2021	Observacional transversal	● SC	“Afeta a conexão entre as CCI, com disfunção nas FBTE, reduzindo a capacidade de percepção da fala em um ambiente ruidoso.”
			● Envelhecimento	
**47**	Edvall et al.^(56)^ -2022	Caso controle	● SC	“Perda da conexão sináptica entre as CCI e as fibras aferentes do NA.”
			● Zumbido	
**48**	Turner et al.^(57)^ -2022	Caso controle	● SC	“Alteração sináptica entre as CCI e as fibras nervosas auditivas.”
			● Zumbido	
**49**	Bramhall et al.^(24)^ -2022	Caso controle	● SC	“Perda das sinapses entre as CCI e as fibras do nervo auditivo aferente.”
			● Exposição ao ruído	

**Legenda:** SC = Sinaptopatia Coclear; PAO = Perda Auditiva Oculta; PAC = Processamento Auditivo Central; PASN = Perda Auditiva Sensorioneural; NA = Nervo Auditivo; CCI = Célula Ciliada Interna; FMTE = Fibras de Média Taxa Espontânea; FBTE = Fibras de Baixa Taxa Espontânea; SA = Sistema Auditivo; CCE = Célula Ciliada Externa.

Quanto ao tipo ou desenho de estudo, verificou-se que 31 (63,2/100%) eram do tipo caso-controle e 36,7% (18/49) eram observacionais transversais.

### Terminologia utilizada e sua aplicação

Dos 49 artigos selecionados, 61,2% (30/49) utilizaram a terminologia SC para designar o fenômeno que estava pesquisando, 4,1% (02/49) utilizaram apenas o termo PAO e 34,7% (17/49) utilizaram os dois termos.

Dos artigos que adotaram a terminologia SC (estudos 3, 7, 10, 11, 12, 14, 15, 16, 18, 19, 20, 21, 22, 23, 25, 26, 29, 31, 32, 33, 35, 37, 43, 44, 45, 46, 47, 48 e 49), as definições mais utilizadas foram as de Kujawa e Liberman^(1)^, Makary et al.^(58)^, Sergeyenko et al.^(59)^ e Liberman e Kujawa^(60)^. Segundo as definições adotadas nestes artigos, a SC seria a perda de sinapses entre as CCI e as fibras do nervo auditivo, que produz lesões em fibras de baixa taxa de descarga espontânea e alto limiar, na ausência de alteração permanente do limiar auditivo^(1)^. Foi também citada a definição de redução na inervação eferente coclear e a perda de sinapses aferentes entre o nervo coclear e as células sensoriais^(59,60)^. Estes autores concordam que a SC tem sido evidenciada em estudos com animais, roedores e primatas, principalmente como consequência da exposição a níveis elevados de intensidade sonora^(1,27-29)^. Devido o comprometimento das sinapses com as fibras eferentes, ocorre comprometimento da codificação do estímulo acústico, uma vez que este inicia o *input* auditivo no Sistema Auditivo Nervoso Central. Com isto, pode-se inferir que os estudos que utilizaram a terminologia SC de fato objetivaram estudar um fenômeno que se restringe a um local específico de lesão ou mecanismos auditivos. Além disso, não há divergências entre os autores em relação à definição desta^(1,58-60)^.

O termo PAO isolado foi utilizado pela minoria dos estudos (estudo 1 e 21). Dentre os dois artigos que o adotaram para designar seu objetivo de estudo, um deles o descreveu como uma condição na qual há alteração no sistema auditivo sem que haja mudança no limiar auditivo (estudo 21). Desta forma, os autores consideram a PAO como um sinal de uma doença auditiva e não como a causa em si. No outro artigo (estudo 1) a PAO foi descrita como a desaferenciação entre as fibras do nervo coclear e o GE, em que os limiares auditivos permanecem dentro da normalidade e existe função prejudicada de fibras eferentes que se projetam do tronco encefálico para a cóclea.

Os termos SC e PAO foram adotados em conjunto nos estudos 2,4, 5, 6, 8, 13, 17, 24, 27, 28, 30, 34, 36, 39, 40, 41 e 42. Dos 17 artigos, 76,5% (13/17) assumem a PAO como sinônimo de SC (estudos 2,4, 5, 6, 13, 24, 27, 30, 34, 36, 39, 41 e 42), utilizando as definições de Schaette e colaboradores^(3)^, Kuwaja e Liberman^(1)^. Enquanto, 23,5% (04/17) os diferenciam quanto ao fenômeno patológico e o sinal deste (estudos 8, 17, 28 e 40). Em outras palavras, estes autores assumem que uma possível causa para a PAO é a SC.

O termo PAO é utilizado como designação genérica para ao menos 14 doenças que acometem o sistema auditivo e promovem queixa de compreensão de fala no ruído na ausência de perda auditiva periférica. Este termo é amplamente aceito para diferentes descrições fisiopatológicas, desde que não haja alterações no limiar auditivo. Por este motivo, é necessária cautela ao associá-lo como sinônimo da SC, que é caracterizada de forma muito específica, principalmente ao que diz respeito ao seu processo fisiopatológico.

Ainda é necessário acrescentar, que durante a busca dos artigos, em dois dos selecionados, observou-se o uso do termo “transtorno do espectro da neuropatia auditiva” fazendo menção a desaferenciação coclear. A neuropatia auditiva é uma condição bem estabelecida, em que há comprometimento do VIII par craniano, devido a alteração de sincronia neural durante a transmissão sináptica. O local de comprometimento do nervo auditivo é variável e pode haver perda auditiva periférica de diferentes graus, unilateral ou bilateral, simétrica ou não. Desta forma, a neuropatia auditiva é uma condição diferente da SC^(61)^.

### População estudada

Os estudos selecionados investigaram diferentes populações e/ou condições. A população exposta a níveis elevados de pressão sonora foi objetivo de pesquisa de 44,8% (22/49) dos estudos, seguida da população com zumbido uni ou bilateral em 24,4% (12/49), 12,2% (06/49) nas condições envelhecimento e 10,2% (05/49) de saúde “hígida”. Perda auditiva condutiva, perda auditiva sensorioneural e Anemia Falciforme representaram 2,0% (01/49) dos estudos. Cabe ressaltar que três artigos estudaram mais de uma condição, dois deles abordaram a exposição a níveis elevados de pressão sonora e zumbido e um artigo as condições de exposição a níveis elevados de pressão sonora e envelhecimento.

A maioria das condições abordadas pelos estudos são apontadas como de risco para a presença da SC (exposição ao ruído, envelhecimento, zumbido). A condição de exposição a níveis elevados de pressão sonora foi a mais estudada (estudos 3, 4, 5, 6, 10, 12, 13, 15, 16, 19, 23, 24, 27, 31, 34, 39, 40, 41, 42, 43, 45, 49), possivelmente por apresentar mecanismos de dano fisiológico conhecidos e relativamente passíveis de controle no que diz respeito ao estudo da SC. E ainda, por ser o fator etiológico mais fundamentado desta patologia. A segunda condição mais investigada foi o zumbido (estudos 1, 7, 8, 9, 11, 13, 30, 31, 36, 38, 47, 48), que também é apontado como um sintoma da SC. Entretanto, algumas considerações quanto a esta condição são necessárias, por se tratar de um sintoma heterogêneo em etiologia, localização, características acústicas, comorbidades associadas, entre outros^(62)^. O zumbido frequentemente é associado a perda auditiva, trauma acústico, exposição a níveis elevados de pressão sonora, uso de ototóxicos, alterações cardiovasculares, alteração temporomandibular ou a ausência de causas aparentes^(31)^. Desta forma, para inferir que o zumbido se deve a SC, outros possíveis fatores devem ser excluídos. Os estudos sobre zumbido incluídos nesta revisão, não referiram excluir ou documentar a presença destas outras condições em sua amostra, com exceção da perda auditiva. O mesmo ocorre para as condições de envelhecimento (estudos 18, 35, 37, 42, 44, 46), de saúde “hígida” (estudos 2, 20, 22, 26, 28, 29) e de queixa de compreensão de fala (14, 25, 32). Para afirmar a presença de SC nestas populações, seria necessária a exclusão das alterações do sistema auditivo nervoso central, pois as mesmas também promovem alterações em habilidades supralimiares^(63)^.

As condições de perda auditiva condutiva (estudo 33), perda auditiva sensorioneural (estudo 17) e de Anemia Falciforme (estudo 21), também foram encontradas na busca do presente estudo. O estudo que investigou indivíduos com perda auditiva condutiva utilizou o termo SC. Entretanto, o estudo apenas indicou que a perda auditiva condutiva crônica em adultos pode ser um risco para o desenvolvimento da sinaptopatia coclear. O estudo sobre perda auditiva sensorioneural utilizou os termos SC e PAO como sinônimos e aplicáveis à condição estudada. Entretanto na SC não há alteração de limiar auditivo^(1)^, desta forma, a designação do se investigava pode ter sido equivocada. Por fim, o estudo de indivíduos com Anemia Falciforme utilizou o termo PAO apenas de forma genérica para indicar alterações no sistema auditivo que não promovem alteração no audiograma.

## CONCLUSÃO

A terminologia SC foi a mais empregada pelos estudos incluídos, sendo que todos eles faziam referência ao processo fisiopatológico de desaferenciação entre as fibras do nervo coclear e as CCI. A maioria dos estudos que adotou ambos os termos, os utilizou como sinônimo, enquanto os outros estudos descreveram a PAO como sendo uma possível consequência da SC. A menor parte dos estudos utilizou apenas o termo PAO, considerando como um sinal de uma alteração auditiva.

## Apêndice 1 Estratégia de busca utilizada de acordo com a base de dados

**Table:**

**Base de dados**	Estratégia de busca
**PUBMED**	(*(“Auditory Synaptopathy”[All Fields] OR ((“neuron s”[All Fields] OR “neuronal”[All Fields] OR “neuronally”[All Fields] OR “neuronals”[All Fields] OR “neurone s”[All Fields] OR “neurones”[All Fields] OR “neuronic”[All Fields] OR “neurons”[MeSH Terms] OR “neurons”[All Fields] OR “neuron”[All Fields] OR “neurone”[All Fields]) AND (“synaptopathies”[All Fields] OR “synaptopathy”[All Fields])) OR “Hidden Hearing Loss”[All Fields]) AND “etiology”[All Fields]) OR “causality”[All Fields] OR “diagnosis”[All Fields].*
**EMBASE**	('auditory synaptopathy'/exp OR 'auditory synaptopathy' OR 'neuronal synaptopathy' OR 'hidden hearing loss'/exp OR 'hidden hearing loss') AND ('etiology'/exp OR 'etiology') OR 'causality'/exp OR 'causality' OR 'diagnosis'/exp OR 'diagnosis'
**CINAHL**	*Auditory Synaptopathy” OR “Neuronal Synaptopathy” OR “Hidden Hearing Loss” AND etiology OR causality OR diagnosis.*
**Web of Science**	*“Auditory Synaptopathy” (All Fields) OR “Neuronal Synaptopathy” (All Fields) OR “Hidden Hearing Loss” (All Fields) AND etiology (All Fields) OR causality (All Fields) OR diagnosis (All Fields).*
